# Cardiac Computed Tomography as an Alternative Technique in the Assessment of Acute Myocardial Ischemia in the Context of COVID-19 Infection

**DOI:** 10.7759/cureus.11415

**Published:** 2020-11-10

**Authors:** Diego Xavier Chango Azanza, Mónica Chapa Ibargüengoitia, Sandra Rosales Uvera

**Affiliations:** 1 Cardiovascular Imaging, National Institute of Medical Sciences and Nutrition Salvador Zubiron, Mexico City, MEX; 2 Cardiovascular Imaging, National Institute of Medical Sciences and Nutrition Salvador Zubiran, Mexico City, MEX

**Keywords:** covid-19 infection, ischemic dilated cardiomyopathy, myocardial infarction, cardiac computed tomography

## Abstract

Coronavirus disease 2019 (COVID-19) has been shown to result in coagulation abnormalities and predisposes patients to thrombotic status, both in the venous and arterial circulations. Herein, we report the case of a 60-year-old patient with COVID-19 pneumonia confirmed by polymerase chain reaction (PCR) who experienced signs and symptoms of myocardial ischemia. A cardiac computed tomography (CT) demonstrated an extensive coronary artery multivessel disease and ischemic dilated cardiomyopathy in a non-invasively approach allowing to define the coronary obstructive involvement in the acute stage of the disease.

## Introduction

Coronavirus disease 2019 (COVID-19) caused by the severe acute respiratory syndrome coronavirus-2 (SARS-CoV-2) has been shown to result in coagulation abnormalities predisposing patients to thrombotic events in the venous and arterial systems [1]. Arterial and venous thromboembolic events are prevalent in patients with severe COVID-19. The incidence of venous thromboembolic events in patients admitted to the intensive care unit (ICU) ranges from 20-35%, and deep venous thrombosis has been identified in 70-100% of patients who died from COVID-19 [2]. Furthermore, arterial thrombosis resulting in stroke and/or myocardial infarction occurs in up to 4% of patients with COVID-19 infection admitted to the intensive care unit [3]. We describe the case of a patient admitted with confirmed COVID-19 pneumonia who presented with signs and symptoms of acute myocardial ischemia. We performed a coronary computed tomography (CT) to determine acute coronary artery disease by a non-invasive approach.

## Case presentation

**Figure 1:**
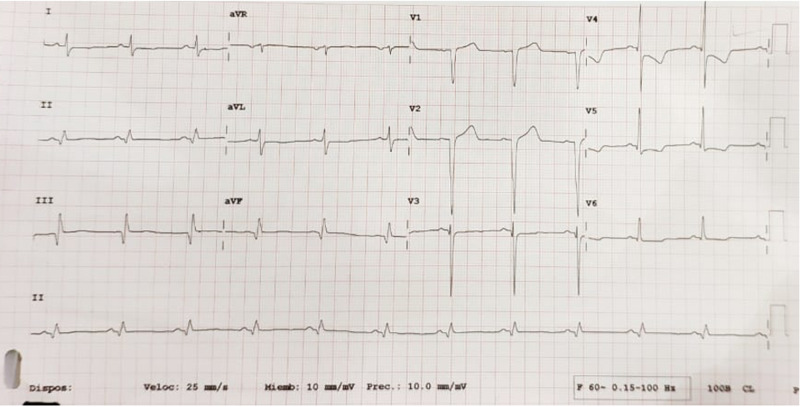
12-lead electrocardiogram of the patient.

A 60-year-old man was admitted to the hospital due to fevers, cough, fatigue, and dyspnea of a few days of duration. He had a medical background of hypertension diagnosed five years ago under medical treatment. He denied a smoking history. He also reported experiencing weight gain and nocturia in the last four months. He had a New York Heart Association (NYHA) class II functional dyspnea without any known coronary artery disease. On admission, he had a positive polymerase chain reaction (PCR) test for COVID-19 infection. He was hemodynamically stable with a blood pressure of 130/80 mmHg, a heart rate of 70 beats per minute (bpm), a temperature of 37.2 degrees Celsius, a saturation of 92% on five liters of oxygen by nasal cannula. Twenty-four hours after hospitalization he suffered typical chest pain with radiation to the left shoulder that improved after a few minutes with the use of vasodilators (intravenous nitroglycerin) without any other episodes of angina. His electrocardiogram showed sinus rhythm, a heart rate of 72 bpm with the presence of pathological ¨Q¨ waves at the inferior leads, lack of ¨R¨ wave voltage in the anterior precordial leads (v1-v3), and ST-T segment abnormalities at the inferolateral precordial leads (v4-v6) (Figure 1).

**Figure 2:**
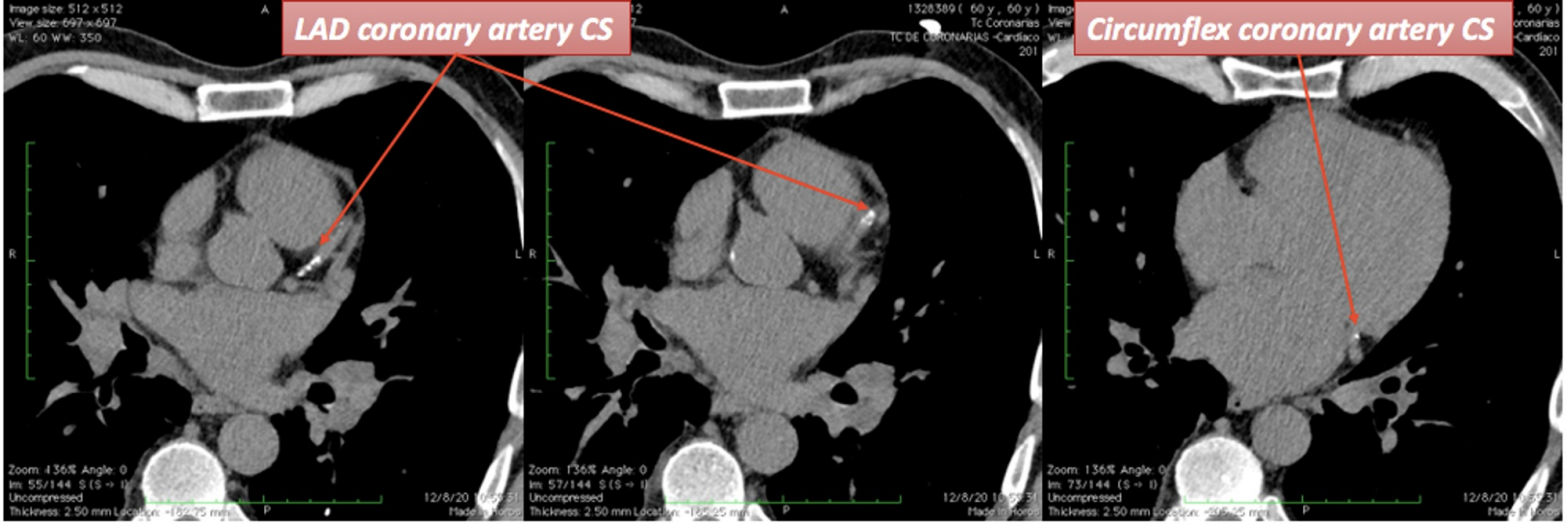
Non-contrast computed tomography coronary artery calcium score. LAD: Left anterior descending artery, CS: calcium score.

Laboratory studies were remarkable for an elevated ultrasensitive troponin I of 6203 pg/ml (reference 0-15 pg/ml), a type B natriuretic peptide of 9000 pg/ml (reference 0-100 pg/ml), a D-dimer of 460 nl/ml (reference 0-250 nl/ml), and a C-reactive protein of 29.8 mg/L (Ref <10 mg/L). Six days after admission and posterior to his clinical improvement we performed a coronary CT angiography to assess for a coronary artery obstruction. Non-contrast cardiac CT images denoted a total coronary artery calcium score of 145 Agatston units (AU) related to a moderate calcification (left anterior descending coronary artery calcium score of 122.9 AU and circumflex coronary artery calcium score of 3.1 AU). We also found a mild ascending aortic artery calcification (Figure 2).

**Figure 3:**
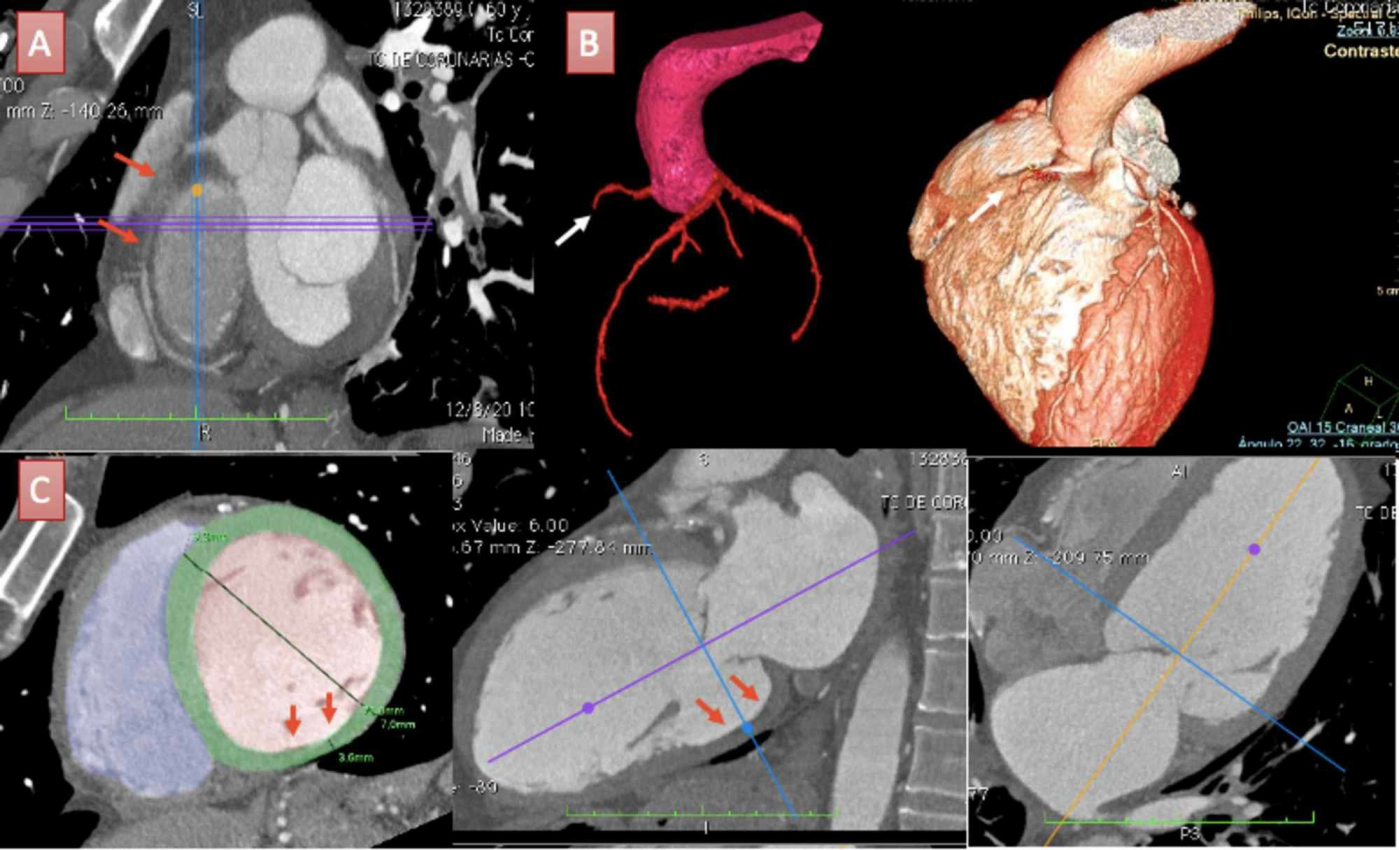
Coronary computed tomographic angiography findings of the patient. A: Multiplane reconstruction of the right coronary artery (RCA) showing total occlusion at the proximal segment (red arrows). B: Tri-dimensional (3D) volume rendering of coronary arteries and cardiac volume showing total occlusion of the RCA (white arrows) and distribution of coronary arteries from an anteroposterior view of the heart. C: Multiple planes reconstruction of the left ventricle (LV). LV short-axis view (bottom left) estimated an end-diastolic LV diameter of 70 mm consistent with a severe dilatation and denoted an inferobasal wall thickness of 3.6 mm (red arrows). LV long-axis two and four-chamber views (bottom middle and right) consistent with inferior basal LV thinned wall (red arrows).

Iodinated contrast images of the coronary CT angiography denoted extensive multivessel coronary artery compromise and the presence of a severe biventricular dilated cardiomyopathy. Both left and right coronary arteries seemed to have a normal origin of its appropriate sinuses of Valsalva. The right coronary artery (RCA) had a total occlusion at the proximal segment and the distal segment was visible due to collateral coronary filling. Basal and medial left ventricular (LV) inferior segments presented thinned and akinetic motion concerning to inferior pathological ¨Q¨ waves in the electrocardiogram in the context of previous myocardial infarction (Figure 3).

**Figure 4:**
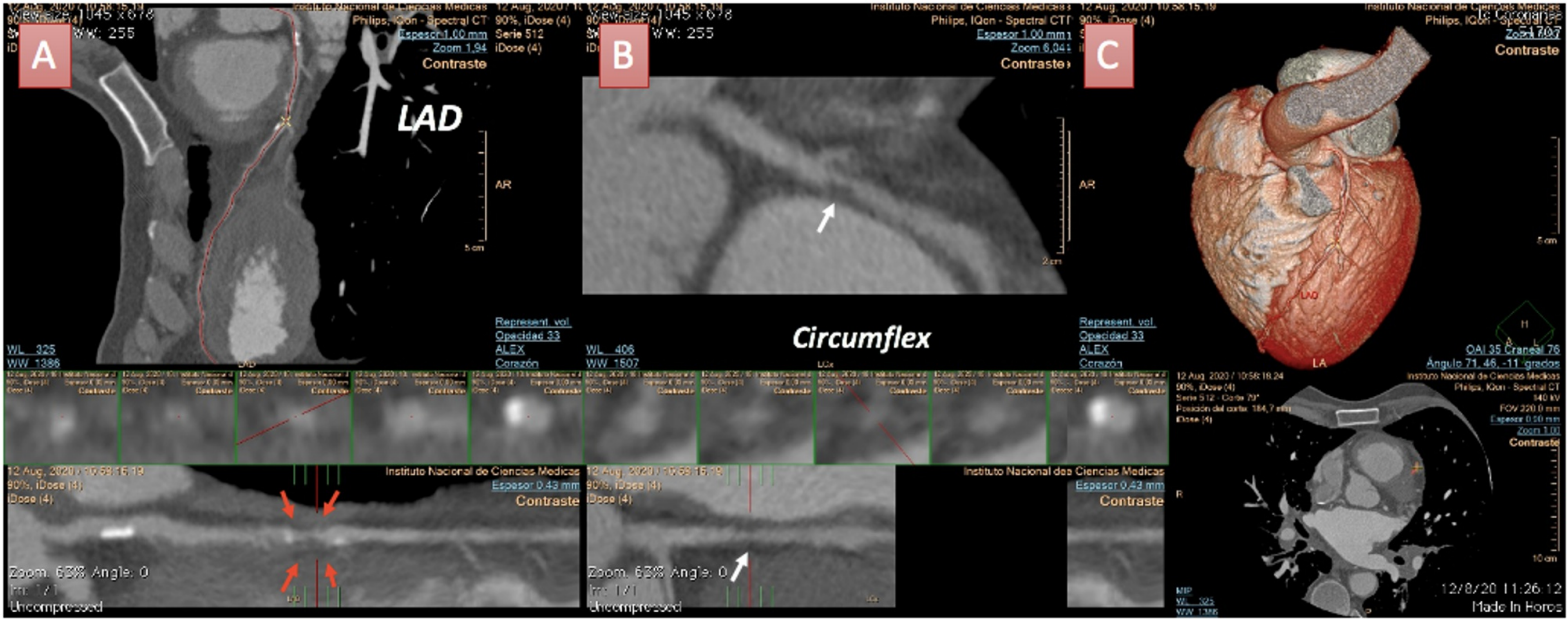
Coronary computed tomographic angiography findings of the patient. A: Left anterior descending (LAD) coronary artery in a curve multiplanar reconstruction (MPR) view with diffuse involvement and calcified plaques. Also, there was a large non-calcified plaque (red arrows) at the medial segment causing a significative obstruction (75-99%). B: Circumflex coronary artery in a curve MPR view showing a non-calcified plaque (white arrow) with non-significant obstruction (25-49%). C: 3D volume rendering of LV and coronary arteries distribution of an anteroposterior view.

The left anterior descending (LAD) coronary artery had extensive involvement with the presence of diffuse non-obstructive calcified plaques but also exhibited a large non-calcified plaque at the medial segment, presenting with characteristics of a vulnerable plaque (positive remodeling with spotty calcifications) causing a significant coronary obstruction (75-99%). The circumflex coronary artery also denoted a non-calcified plaque at the medial segment causing a non-significant obstruction (25-49%) (Figure 4). 

Data analysis of cardiac CT angiography retrospective acquisition also allowed us to define the functional assessment of the volumes and biventricular function. We determined a severe biventricular dilatation and systolic dysfunction in the context of ischemic cardiomyopathy (Table 1, Video 1).

**Table 1:** Functional parameters of cardiac CT assessment of the patient. LV: left ventricle, RV: right ventricle. EDV: end-diastolic volume. ESV: end-systolic volume. SV: stroke volume. LVEF: left ventricular ejection fraction. RVEF: right ventricular ejection fraction.

Functional parameters	Value
LV EDV	325ml (indexed 178ml/m2)
LV ESV	260ml (indexed 142ml/m2)
LVEF	20%
LV SV	65ml (indexed 35ml/m2)
RV EDV	177ml (indexed 97ml/m2)
RV EV	140ml (indexed 76,9ml/m2)
RVEF	20%

 

**Video 1:** Cardiac functional computed tomography assessment of the patient. Multiplanar reconstructions of the heart at the short axis and long-axis views.

This case provides evidence of the usefulness of cardiac CT imaging in determining the presence of myocardial ischemia in the context of an acute COVID-19 infection. This patient also had signs and symptoms of heart failure four months before the infection without receiving any further evaluation of his previous cardiac condition. We found an extensive multivessel coronary artery disease associated with ischemic cardiomyopathy and severe biventricular systolic dysfunction. This patient recovered from his active infection successfully. We recommended a cardiovascular team approach to decide the best option for coronary artery revascularization. 

## Discussion

It has been recently proposed that severe COVID-19 infection is a microvascular disease in which the coronavirus activates endothelial cells triggering exocytosis with a rapid vascular response that drives microvascular inflammation and thrombosis [3]. These effects are believed to be secondary to inflammation, platelet activation, endothelial dysfunction, and venous stasis [4]. Patients with severe COVID-19 infection often have laboratory findings consistent with a hypercoagulable state suggesting widespread thrombosis and fibrinolysis as well as elevated levels of D-dimer, Von Willebrand factor (VWF), and Factor VIII. These patients also manifest a hyper-inflammatory state or “cytokine storm” characterized by elevated levels of inflammatory markers such as the C-reactive protein (CRP) and interleukin-6 which have been linked to the severity of pneumonia and mortality [3]. Autopsy studies have suggested that both endothelial inflammation and microvascular thrombosis are prominent, with inflammatory cells attached to the endothelium of small vessels in the lung, kidney, heart, and liver [5]. Nevertheless, we have seen fewer cases of patients with ST-elevation myocardial infarction (STEMI) and other thrombotic disorders such as cerebrovascular accidents during the pandemic which could be mainly a result of the social isolation and behavioral changes. Furthermore, emergency cardiac catheterization reveals a variety of findings in COVID-19 patients with ST-elevation, including classic type 1 myocardial infarction (obstructive coronary artery disease), angiographically normal epicardial coronary arteries, and/or left ventricular dysfunction due to myocarditis or stress-induced cardiomyopathy [6,7]. A recently single-center, observational study of 115 consecutive ST-segment elevation myocardial infarction (STEMI) patients with and without COVID-19 disease treated with primary percutaneous coronary intervention showed that STEMI patients presenting with concurrent COVID-19 infection had significantly higher rates of multi-vessel thrombosis, stent thrombosis, higher modified thrombus grade post first device, increased use of glycoprotein IIb/IIIa inhibitors and thrombus aspiration, elevated D-dimers, and lower myocardial blush grade and left ventricular function [8].

Coronary CT angiography could offer a useful alternative for the study of these patients to avoid the spread of the virus during invasive catheterization in specific situations. The recently published guidelines for the management of acute coronary syndromes in patients presenting without persistent ST-segment elevation indicate that this technique is an alternative for invasive coronary angiography when there is a low-to-intermediate likelihood of coronary artery disease and when troponin and/or ECG are normal or inconclusive with a strong recommendation (IA) [9]. There is emerging evidence about the high negative predictive value of coronary CT angiography in this context where the absence of coronary obstruction was present in 26% of patients [10]. This technique also allows qualitative and quantitative assessment of atherosclerotic plaque burden that can be used to measure the burden of calcified, noncalcified, and low-attenuation plaque, as well as the total coronary plaque burden providing important prognostic information. In a study, a low-attenuation plaque was the strongest predictor of fatal or nonfatal myocardial infarction, exceeding other established markers including cardiovascular risk scores, computed tomography calcium scoring, and coronary artery stenosis. Patients with a low-attenuation plaque burden >4% were five times more likely to suffer a fatal or nonfatal myocardial infarction [11].

## Conclusions

COVID-19 infection is related to endothelial damage that can precipitate embolic events promoting an acute myocardial infarction and cardiac computed tomography represents a valuable alternative for evaluating these patients achieving an anatomical and functional status assessment to develop further therapeutic strategies and trying to avoid the virus spread during invasive coronary angiography. Characterization of plaque burden can be useful to determine those patients at risk of acute coronary syndrome in this context and individualized patient assessment is necessary to decide the best clinical decision making.
